# Selective synthesis of substituted amino-quinoline derivatives by C-H activation and fluorescence evaluation of their lipophilicity-responsive properties

**DOI:** 10.1038/s41598-019-53882-z

**Published:** 2019-11-27

**Authors:** Yasufumi Fuchi, Masaomi Sakuma, Kohei Ohyama, Ryusuke Hagihara, Minaki Kohno, Koichi Hamada, Akihiro Mizutani, Satoru Karasawa

**Affiliations:** 1 Faculty of Pharmaceutical Sciences, Showa Pharmaceutical University, 3-3165 Higashi-tamagawagakuen, Machida, 194-8543 Japan; 2 Graduate School of Pharmaceutical Sciences, Kyushu University, 3-1-1 Maidashi, Higashi-Ku, Fukuoka, 812-8582 Japan; 3 PRESTO, Japan Science and Technology Agency, Kawaguchi, 332-0012 Japan

**Keywords:** Fluorescent probes, Single-molecule fluorescence, Reaction mechanisms, Lipids, X-ray diffraction

## Abstract

Push-pull type fluorescent amino-quinoline derivatives (TFMAQ) bearing phenyl aromatic groups in the 8-position (TFMAQ-8Ar series) were synthesized via palladium-catalyzed C-H activation reaction in short steps. The *N*-arylation or C-H activation reactions were selectively controlled with high yield by combinations of palladium and phosphine ligands. The TFMAQ-8Ar analogues exhibited fluorescent solvatochromism in non-polar and polar solvents. In non-polar solvent, the absolute fluorescence quantum yield was high, wheareas the fluorescence was almost quenched in polar solvent. The TFMAQ-8Ar derivatives also showed high fluorescence emission at solid state owing to the planar structure between the quinoline ring and phenyl ring at the 7-amino group, as demonstrated by X-ray crystal structure analysis. The fluorescence imaging of 3T3-L1 cell using TFMAQ-8Ar derivatives was performed by confocal laser microscopy. Strong and specific emissions at lipid droplets were observed owing to the accumulation of TFMAQ-8Ar derivatives. Therefore, we propose that the TFMAQ-8Ar derivatives should become a versatile fluorescence probe for the live imaging of lipid droplets.

## Introduction

Fluorescence molecules with electron-donor groups and electron-withdrawing groups in a π-conjugated system are used in some biological analysis^1,2^. Nitrobenzoxadiazole derivatives and dansyl derivatives are representative push-pull type fluorescence groups with a bicyclic skeleton^3–5^. Such molecules with both donor and acceptor groups in an aromatic ring generally have a solvatochromic effect on UV-Vis absorption and the fluorescence emission of the solvent environment along with changes in the intramolecular charge transfer (ICT) state^6,7^. By using the fluorescence emission shift in various solvents, environment-responsive fluorescent probes are developed for multicolour imaging sensors^8–10^. Nile Red is one of the environment-sensitive fluorescence probes that exhibit a bathochromic shift in polar solvents^11–13^. In cellular imaging, it shows red fluorescence emissions in cytoplasmic membranes containing polar phospholipids, whereas green fluorescence emissions are detected in neutral lipids such as cholesterols and triacylglycerols^3,14^. Lipid droplets (LDs) consisting of neutral lipids are essentially regarded as fat storage organelles in lipid metabolic systems. The various roles of LDs in cells have been increasingly investigated such as hepatitis C virus production^15^, and early embryonic^16^ and cancer development^17^. Recently fluorescent probes^18–20^ other than Nile Red as well as BODIPY 493/503^21^ for staining cellular LDs have been developed as an adipose assay tool. These probes exhibited efficient emission and selective fluorescence signals for intracellular LDs; however, multistep synthesis was required to construct the framework.

**Figure 1:**
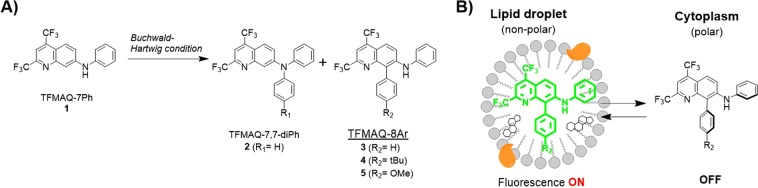
(**A**) C8-H functionalization of TFMAQ by Buchwald–Hartwig reaction conditions. (**B**) Specific fluorescence imaging of lipid droplets using TFMAQ-8Ar compounds.

Our group previously synthesized bicyclic push-pull type fluorescence amino-quinoline (TFMAQ) derivatives, which have bis-trifluoromethyl groups and an amino group in a quinoline ring using a few steps and studied their unique fluorescence properties in a solution state and a solid state^22^. We reported that TFMAQ exhibited fluorescence colour changes depending on solvent polarity and crystalline phase transition. The TFMAQ chromophore was further applied to thermo-responsive nanomaterials as *in vivo* tumour-imaging agent^23,24^. The TFMAQs were also derivatized to phenyl aromatic-substituted amine analogues to investigate the photophysical properties at solid state^25,26^. These derivatives were found to have some crystal polymorphs, and emitted fluorescence by external stimuli such as grinding at solid state accompanied by conformational changes. From these reports, it has been suggested that simple phenyl group as N7-substituent is suitable for higher fluorescence quantum yield of TFMAQ chromophore in solution states. In the synthesis of TFMAQ-7,7-diAr compound (**2**) from TFMAQ-7Ar (**1**), we discovered an interesting side-product, in which 8-position C-H was substituted by an aromatic group (TFMAQ-7,8-diPh, **3**) despite non-substitution in the N7-position when conducted under Buchwald–Hartwig cross-coupling reaction conditions (Fig. 1A). Although several examples of C8-functionalization of quinoline have been reported such as Rh-catalyzed reaction^27^ and C-H arylation of quinoline *N*-oxide^28^, the Pd-catalyzed C8-H direct arylation in TFMAQ series is a significantly unique reaction that requires further derivatization. Since the aryl group in the TFMAQ 8-position is expected to be twisted against the quinoline plane, the fluorescence properties will shift depending on the twisted-intramolecular charge transfer (TICT) state^29,30^. Herein, we report the efficient synthesis and photophysical properties of novel fluorescent TFMAQ-8Ar compounds (**3**–**5**) in solution and solid state. In addition, by applying the lipophilicity-responsive fluorescence property of TFMAQ-8Ar compounds, cellular fluorescence imaging of differentiated 3T3-L1 cells was performed to detect intracellular LDs (Fig. 1B).

## Result and Discussion

### Investigation of C-H activation reaction

To investigate the C-H activation reaction of TFMAQ derivatives, we performed several Buchwald–Hartwig reactions for compound **1**. The TFMAQ-7Ph (**1**) was synthesized in two steps from 1,3-diaminobenzene and hexafluoroacetylacetone according to our report in literature^22^. In preliminary experiments related to the solvent and base (Table S1), toluene and potassium *tert*-butoxide was the optimal combination to obtain C-H activated product **3** using bromobenzene as coupling reagent, palladium acetate and DPPF (1,1′-Bis(diphenylphosphino)ferrocene) as catalyst, and a phosphine ligand. As given in entry 1 (Table 1) at primary conditions of 110 °C for 3 h, TFMAQ-8Ph **3** was obtained with 30% yield instead of the normal amino-coupling product TFMAQ-7,7-diPh (**2**). We then examined some phosphine ligands in the presence of palladium acetate (entries 2–7). By varying phosphine compounds with monodentate or bidentate ligands such as XPhos, Xantphos, and JohnPhos, compound **2** was selectively obtained with higher yield (77–86%), while C-H activated product **3** was observed with trace yield. As these ligands are usually applicable for Buchwald–Hartwig cross-coupling, the reaction from compound **1** proceeded via a well-known catalytic cycle consisting of Pd^0^/Pd^II^ oxidation state^31^ to produce compound **2**. Although the reaction using DPPF ligand in a type of ferrocene achieved selective synthesis for C-H activated product **3**, in the case other ferrocene ligands such as DtBPF and QPhos, compound **2** was obtained selectively with high yield (73% and 81% in entry 7 and 8, respectively). These results suggest that the ferrocenyl group is not an essential functional group in the C-H activation reaction of TFMAQ-7Ph (**1**). Next, we studied other palladium sources in this reaction. When using the divalent palladium complex Pd(DPPF)Cl_2_ without the addition of a phosphine ligand, the reaction proceeded selectively to C-H activated product **3** with higher yield (88%, entry 9). In the case of Pd(PPh_3_)Cl_2_, the C-H activation reaction also proceeded selectively (61%, entry 10). The selectivity and yield using Pd(PCy_3_)Cl_2_ were slightly lower than the other divalent palladium-phosphine complexes (12% for **2** and 40% for **3** in entry 11). These results indicate that the divalent palladium complex preferentially catalyses the C-H activation reaction instead of the 7*N*-arylation of TFMAQ-7Ph **1**.

**Table 1:** Reaction screening of C-H activation for TFMAQ-7Ph **1** catalyzed by palladium-ligand systems.


Entry^a^	1	2	3	4	5	6
[Pd]	Pd(OAc)_2_	Pd(OAc)_2_	Pd(OAc)_2_	Pd(OAc)_2_	Pd(OAc)_2_	Pd(OAc)_2_
Ligand	DPPF	*t*-Bu_3_P	DPPE	XPhos	Xantphos	JohnPhos
**2** yield^b^ (%)	Trace^c^	77	n.d.	63	56	86
**3** yield^b^ (%)	30	n.d.^d^	Trace	Trace	Trace	Trace
**Entry**	**7**	**8**	**9**	**10**	**11**	**12**
[Pd]	Pd(OAc)_2_	Pd(OAc)_2_	Pd(DPPF)Cl_2_	Pd(PPh_3_)_2_Cl_2_	Pd(PCy_3_)_2_Cl_2_	Pd(CH_3_CN)_2_Cl_2_
Ligand	DtBPF	QPhos				
**2** yield (%)	73	81	Trace	Trace	12	n.d.
**3** yield (%)	Trace	Trace	88	61	40	n.d.

^a^The reaction was performed with 0.07 mmol of **1**, 3 eq of PhBr, 18 mol% of palladium [Pd], 30 mol% of phosphine ligand, and 3 eq of *t*-BuOK in 1 mL of toluene at 110 °C for 3 h. ^b^Isolated yield. ^c^Detected only on TLC. ^d^Not detected. DPPE = Ethylenebis(diphenylphosphine). XPhos = 2-Dicyclohexylphosphino-2′,4′,6′-triisopropylbiphenyl. Xantphos = 4,5-Bis(diphenylphosphino)-9,9-dimethylxanthene. JohnPhos = 2-(Di-tert-butylphosphino)biphenyl. DtBPF = Bis[(di-*tert*-butylphosphino)cyclopentadienyl]iron. QPhos = 1,2,3,4,5-Pentaphenyl-1′-(di-*tert*-butylphosphino)ferrocene.

**Figure 2:**
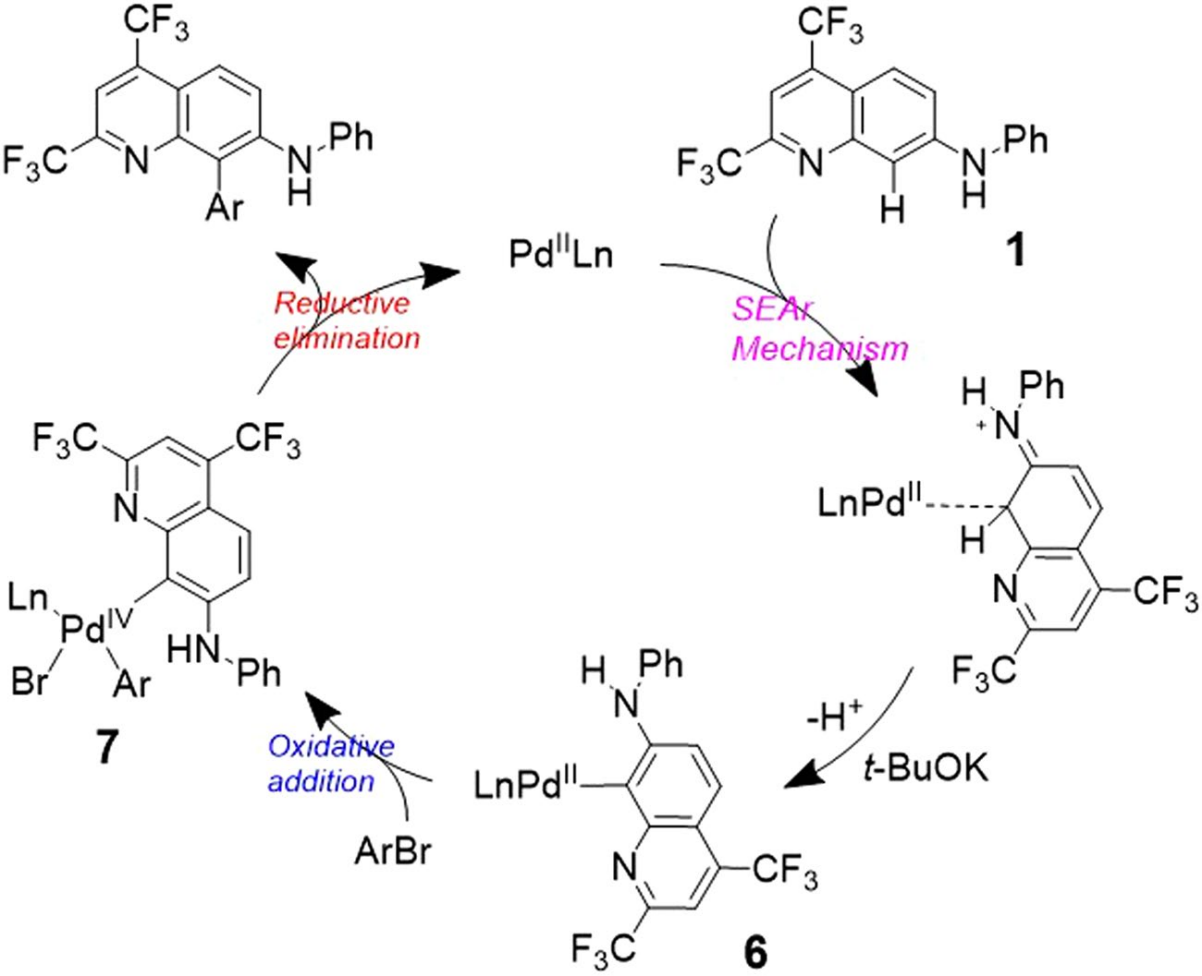
Proposed Pd catalysed C-H activation mechanism of TFMAQ-7Ph.

By applying this reaction condition (entry 9), we synthesized TFMAQ-8Ar compounds **4** (Ar = *p*-*t*BuPh) and **5** (Ar = *p*-MeOPh) with high yields (77% and 80%, respectively) using 4-*tert*-butyl bromobenzene or 4-methoxy bromobenzene as coupling reagents, which have electron donor groups at the para-position. Although further derivatizations were required, this is the optimal reaction condition to obtain C-H activated TFMAQ-8Ar derivatives. The reaction can summarized as follows: palladium (II) acetate with phosphine ligand systems, which can generate Pd(0) *in situ*, catalyses the *N*-arylation, whereas Pd(II)-phosphine complex systems provide the C-H activation product. In addition, substituted quinoline at C8-position derivatives were recently synthesized by electrophilic nitration^32^. Based on these considerations on C-H activations catalysed by palladium species^33–35^, a plausible catalytic cycle for C8-H activation of TFMAQ-7Ph was proposed as follows (Fig. 2). In the first step, the C8-position of quinoline coordinates to Pd(II) through electrophilic aromatic substitution (SEAr). The C8-metalation species **6** deprotonated by a base induces the oxidative addition of aryl bromide to provide Pd(IV) species **7**. Subsequently, it undergoes reductive elimination resulting in the C-H activation product associated with the regeneration of Pd(II) continuing the catalytic cycle. In this case, the possibility of Pd(0)/Pd(II) catalytic cycle could be ruled out because the *N*-arylation product was only observed under conditions using palladium acetate with phosphine ligands (entries 2–8 in Table 1). As a result, we achieved the highly selective and efficient synthesis of either *N*-arylation products or C-H activated products as novel TFMAQ derivatives.

### X-ray crystal structure analyses

The molecular structures of TFMAQ-8Ar compounds are identified by not only NMR spectra and ESI-MS but also X-ray crystal structure analysis. The suitable single crystal of compound **3**–**5** was obtained by recrystallization according to the experimental procedure. The crystallographic data of TFMAQ-8Ar compounds are summarized in Table 1, and ORTEP drawings are shown in Fig. S1. Other detailed parameters are described in the supplementary information (Table S2). In the crystal of compound **4**, two crystallographically independent molecules were observed.

**Figure 3:**
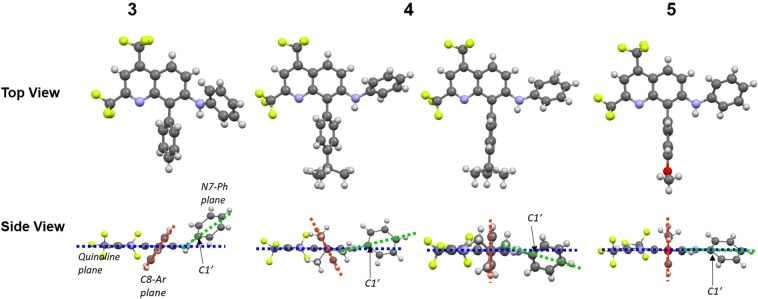
Top-view and side-view structures of compounds **3**–**5** obtained from X-ray crystal structure analyses. Blue, orange, and green dotted lines denote quinoline, 8-phenyl, and 7-phenyl plane, respectively. The C1’ was positioned on the root of N7-phenyl group in each structure.

The distance between the C8-position and the benzene ring of compound **3** was slightly longer (1.502 Å) than that of compounds **4** (1.494 Å) and **5** (1.493 Å). The distance between the C7-position and the N-atom of compound **3** was also longer (1.410 Å) than that of compounds **4** and **5** (≈1.38 Å). Differences in torsion angles were observed in the N7-phenyl ring or C8-phenyl group for the quinoline ring (Fig. 3). The torsion angles of structure **3** and one of structure **4** were 59.40° and 61.60°, respectively, between the C8-aryl and quinoline ring, showing twisted structure toward the right or left side (Table 2 and Fig. 3). In contrast, almost orthogonal angles between C8-aryl groups and the quinoline ring were observed in another structure of compound **4** (89.18°) and compound **5** (89.77°). The torsion angles of the N7-phenyl ring were also twisted toward the quinoline plane in compound **3** and both structures of **4** (64.06° and 17.85° or 16.50°), whereas the lowest value (0.33°) was found in structure **5**. The difference in torsion angles was reflected by the through-spaced short contact between C1’ and the plane extrapolated by the quinoline ring, showing 1.040, 0.328, and 0.096 Å for compounds **3**, **4**, and **5**, respectively. In compounds **3** and **4**, the short intermolecular contacts between H-atoms in the amine group and C-atoms in the N7-phenyl ring were observed as approximately 2.8 Å, which is based on the CH-π interaction; the corresponding contact was missing in compound **5** (Fig. S4B). These results indicate that the planarity between the quinoline ring and N7-phenyl ring increased in the order of structure **3** < **4** < **5**, associated with the increased orthogonality of the structure between the quinoline ring and C8-phenyl ring.

**Table 2:** The selective crystal data of TFMAQ-8-Ar compounds from X-ray structural analyses.

	3	4*	5
*Space Group*	*P*2_1_/*c* (#14)	*C*2/*c* (#15)	*P*-1 (#2)
*Bond length (Å) of C8-Aryl*	1.502	1.494 and 1.490	1.493
*Bond length (Å) of C7-Ph*	1.410	1.380 and 1.376	1.378
*Torsion angle (deg.) of Q-8Ar*	59.40	61.60 and 89.18	89.77
*Torsion angle (deg.) of Q-7Ph*	64.06	17.85 and 16.50	0.33

*Two crystallographically independent molecules were observed.

### Photophysical properties

**Figure 4:**
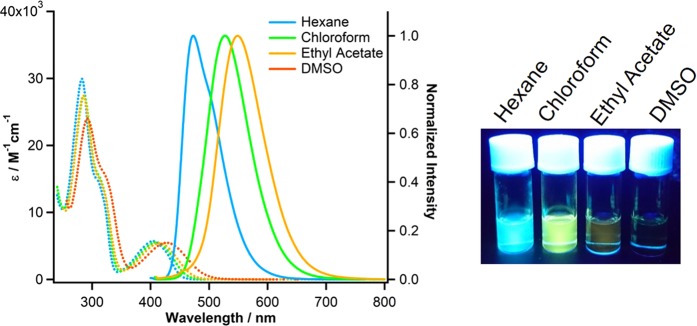
Emission (solid line) and absorption (dotted line) spectra of compound **3** solutions in each solvent (*n*-Hexane, Chloroform, Ethyl Acetate, DMSO). The emission spectrum in DMSO was not detected. Photograph taken under UV light (365 nm) in each solution.

The absorption and emission properties of compounds **3**, **4**, and **5** were investigated in non-polar solvents and polar organic solvents (*n*-hexane, chloroform, ethyl acetate, and DMSO) at room temperature. The fluorescence emission spectra in the solution and solid state were also obtained at room temperature excited at the absorption maxima (400–420 nm). The absorption and emission spectra of compound **3** in various solutions are shown in Fig. 4, while that of compounds **4** and **5** are shown in Figs S2 and S3 (supporting information). The absolute fluorescence quantum yields (*ϕ*) of all compounds in solution state and solid state were also obtained, and are summarized in Table 3 together with comparison of the photophysical data of compound **1**. The fluorescence lifetime (*τ* values) of compounds **3**–**5** were also estimated in each solvent (Table S3).

**Table 3:** Photophysical data of compounds **1**, **3**, **4**, and **5** in the solution.

	1^a^				3				4				5			
	Hex	CHCl_3_	AcOEt	DMSO	Hex	CHCl_3_	AcOEt	DMSO	Hex	CHCl_3_	AcOEt	DMSO	Hex	CHCl_3_	AcOEt	DMSO
λ^abs^_max_/nm	392	401	410	415	404	409	415	428	404	409	413	428	407	412	421	432
λ^FL^_max_/nm^b^	446	504	552	607	473	527	549	n.d.	485	537	558	593	505	537	556	n.d.
*Φ*_fl_	0.48	0.32	0.01	<0.01	0.51	0.54	0.03	n.d.	0.38	0.33	0.06	0.01	0.57	0.42	0.12	n.d.

^a^Ref.^22^. ^b^Excitation at a maximum of the corresponding excited spectra under individual conditions. n.d. = not detected.

According to the absorption spectra of compound **3** in the solutions, the absorption maxima values in *n*-hexane, chloroform, ethyl acetate, and DMSO were 404, 409, 415, and 428 nm, respectively, showing a small red-shift with dependence on solvent polarity, and the extension of π-conjugated system at the 8-position of the quinoline ring compared with TFMAQ-7Ph (**1**). The absorption maxima values of compound **4** in the four solvents were 404, 409, 413, and 428 nm, respectively. These are similar to those of compound **3**, indicating minimal impact from the substituted *tert*-butyl group. In the absorption spectra of compound **5** (λ^abs^_max_ = 407, 412, 421 and 432 nm), small red-shifts were observed in the four solutions compared with compounds **3** and **4**, suggesting the electron-donating effect of the methoxy group.

The emission spectra of compounds **3**–**5** were observed in the range 470–590 nm, irradiated at approximately 400 nm. In non-polar *n*-hexane solution, compounds **3**, **4**, and **5** exhibited broad emissions of approximately 473, 485, and 505 nm, respectively, which shifted longer wavelengths than compound **1** solution (446 nm). These bathochromic shifts were due to the extension of π-conjugated system at the 8-position of the fluorescence quinoline ring. The maximum fluorescence emission of compounds **3**–**5** in chloroform solutions were 527 and 537, also resulting in red-shift compared with compound **1** solution. On the other hand, comparable emission wavelength for compound **1** was observed in a small amount of polar ethyl acetate solution of compounds **3**, **4**, and **5** (549, 558, and 556, respectively). In polar DMSO solutions, a longer wavelength of fluorescence emission was observed only for compound **4** (593 nm) with low *ϕ* value (0.01). Accordingly, higher *ϕ* values were obtained in non-polar *n*-hexane solution (0.51, 0.38, and 0.57 for **3**, **4**, and **5**) and chloroform solution (0.54, 0.33, and 0.42 for **3**, **4**, and **5**). The ethyl acetate solutions exhibited relatively lower *ϕ* values (0.03, 0.06, and 0.12 for **3**, **4**, and **5**) due to its slightly higher polarity. In DMSO solution, the fluorescence spectrum was hard to detect owing to the low *ϕ* values ( < 0.01).

The *τ* values in each solution were obtained to analyse the first-order decay (Table S3). The *τ* values of compound **3** in each solution decreased in the order of *n*-hexane (14.8 ns) > chloroform (8.66 ns) > ethyl acetate (1.27 ns) with increasing solvent polarity. These values are greater than those of compound **1**, suggesting that the excited state of TFMAQ-8Ar was stabilized by the suppression of *syn*-*anti* conformational rotation at the N7-phenyl group owing to the C8-phenyl group. The *τ* values of compounds **4** and **5** in each solution were slightly longer than those of compound **3**. From the *ϕ* values and *τ* values of the photophysical data, radiative rate constants (*k*_*r*_) and non-radiative decay constants (*k*_*nr*_) were determined, respectively. The *k*_*r*_ values of compound **3** in several solvents resulted in little difference (0.024–0.062 ns^−1^). However, the *k*_*nr*_ values of compound **3** showed large differences with increasing solvent polarity (0.033–0.764 ns^−1^). Similar properties were observed in compound **4** (*k*_*r*_ = 0.024–0.029 ns^−1^, *k*_*nr*_ = 0.040–0.347 ns^−1^) and **5** (*k*_*r*_ = 0.023–0.034 ns^−1^, *k*_*nr*_ = 0.026–0.172 ns^−1^). These results indicate that the environment around the chromophore controls the non-radiative decay of fluorescence emissions. In other words, the TICT state (‘fluorescence quenching’ state, Scheme S1) was induced by more polar solvents like typical environment-responsive fluorescence groups^4^. These spectral experiments in the solution states show that the new series of TFMAQs are extremely sensitive to the lipophilic environment owing to the large differences in *ϕ* values between non-polar and polar solutions (e.g. < 0.01 and 0.57 for compound **5**).

The emission spectra and *ϕ* values of compounds **3**–**5** in solid state are shown in Fig. S4A and Table S4. The maximum emission length increased in the order of **3** (493 nm) < **4** (512 nm) < **5** (526 nm) excited at 400 nm. This order is notably consistent with that of the shorter distance between 1’-carbon on N7-phenyl ring and quinoline plane measured by X-ray crystal structure analyses. Therefore, the emission wavelength in the solid state is dominated by increased planarity between the quinoline ring and N7-phenyl ring, associated with the extension of the π-conjugated plane. The *ϕ* values in solid state also increased in the order of **3** (0.11) < **4** (0.19) < **5** (0.38). The *ϕ* values in the solid state is often influenced by the intermolecular distance based on a hydrogen bond, π-π stacking, etc^22^. The order of *ϕ* values among compounds **3**–**5** is consistent with the short intermolecular contact between H-atoms in the amine group and C-atoms in the N7-phenyl ring (Fig. S4B). This suggests that compound **5** without short contact emitted strongly compared with **3** and **4** with short contact.

For the theoretical interpretation of electron states, time-dependent (TD)-DFT calculation at the excited state was performed on the basis of B3LYP/6-31++G (d, p) level. The highest occupied molecular orbital (HOMO) and lowest unoccupied molecular orbital (LUMO) for each compound are shown in Fig. S5. The HOMO orbitals of compound **3** localized near the *N*7-position as an electron-donor site, whereas the LUMO orbitals existed near the trifluoromethyl groups at the quinoline ring as electron-acceptor sites. In the HOMOs, the orbitals of the 8-phenyl group were observed at a low level, suggesting little contribution as an electron-donor for the fluorescent quinoline ring. The *N*-phenyl group is typically regarded as an electron-donor for the fluorescence group, which could form the TICT state^36,37^. In the case of TFMAQ-8Ar series, an aryl group in the 8-position adjacent to the quinoline ring could also be involved in TICT formation. As compound **5** displayed larger distribution of HOMO orbitals at the 8-position anisole ring owing to its methoxy group, compound **4** had HOMO orbitals in the 8-position phenyl ring as large as that of compound **3**. The theoretical calculation results indicate that the high sensitivity of TFMAQ-8Ar compounds to lipophilic environments is caused by an additional twisting group of 8-position aryl rings for the quinoline chromophore.

### Fluorescence imaging of lipid droplet in live cells

**Figure 5:**
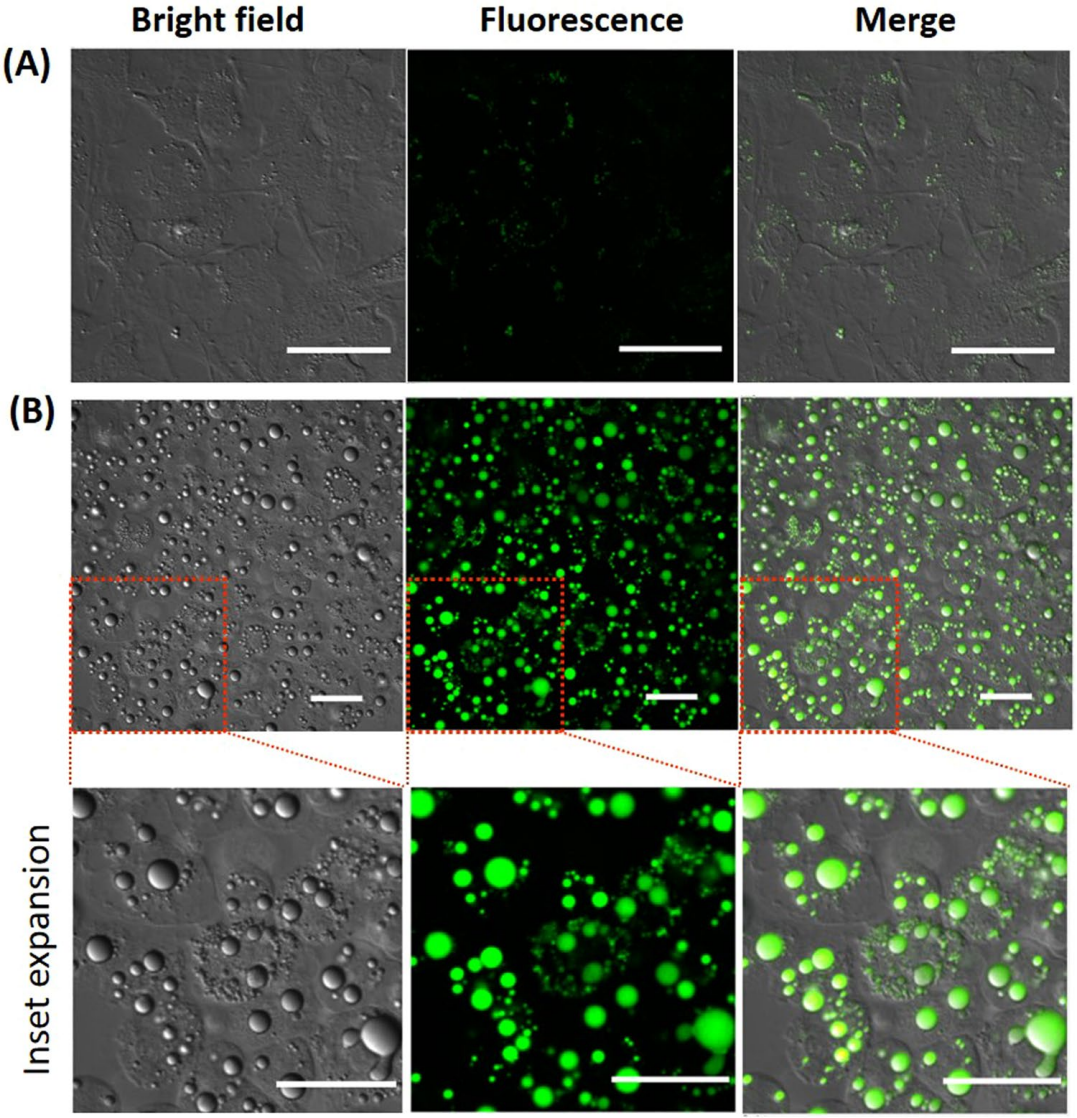
3T3-L1 cell images by confocal laser microscopy staining with compound **3** (1 μM in cultured medium) before **(A)** and after **(B)** differentiation induction. Scale bars = 50 μm.

According to the fluorescence properties described above, new TFMAQ derivatives of **3**–**5** can emit only in non-polar environments with high quantum yield. These characteristics were applied to a fluorescent probe for the specific detection of non-polar environments with high S/N ratio. We performed the fluorescence imaging of LDs containing neutral lipids such as cholesterols and triacylglycerols. 3T3-L1 cells were used for LD imaging after differentiation induction was performed to afford adipocytes. Differentiated 3T3-L1 cells as adipose cells are known to form large size LDs in the cytoplasm^38^. These cells were incubated with 1 μM of compounds **3**–**5** in medium, and then fluorescence images were readily acquired by confocal laser microscopy without washout (excitation: 403 nm, emission: 525 nm). Before the treatment of differentiation induction, weak fluorescence signals were observed within 3T3-L1 cells (Fig. 5A). These signals might be small LDs in normal cells. We also demonstrated the fluorescence imaging of compound **3** in HeLa cells by co-staining with fluorescence probes for the mitochondria or lysosomes. These organelle probes presented few accordant fluorescence signals with TFMAQ-8Ph (**3**), as indicated by scatter diagrams and low values of Pearson’s correlation coefficient^39^ (Fig. S6). In differentiated 3T3-L1 cells, a number of spherical clusters, which are consistent with bloated LDs in published literature^14^, were observed in the bright field (Fig. 5B). The fluorescence signals of compound **3** existed specifically on these spherical droplets without non-specific signals in cells such as membranes and so on. Compounds **4** and **5** were also applied for fluorescence imaging of LDs on differentiated 3T3-L1 cells (Fig. S7). Compound **5** displayed specific fluorescence signals on LD moieties under the same conditions as compound **3** (Fig. S7B). However, significantly weak fluorescence signals were observed in the image of compound **4** (Fig. S7A). This result suggests that compound **4** is not sufficiently soluble in aqueous solution owing to the hydrophobic *tert*-butyl group, or has the potential to bind with some constituents in the medium. When fluorescence imaging was performed using higher concentration of compound **4** (10 μM), the fluorescence on the LDs were observed (Fig. S7C). Furthermore, compound **3** and **5** exhibited the additional utility because specific fluorescence signals were also observed under lower concentration (0.1 μM, Fig. S7D,E) Thus, detection limits of compound **3**, **4** and **5** in fluorescence imaging of LDs are suggested to be 0.1, 10 and 0.1 μM, respectively.

We also investigated the photostability and cell-growth inhibition activity of compounds **3**–**5**, which are properties required for live-cell imaging (Fig. S8). Photoreactions in methanol solution were performed using a high-pressure mercury lamp at room temperature, and the decrease in visible-absorbance was observed for 60 min. From the absorbance decay plots, TFMAQ-8Ar derivatives (**3**–**5**) exhibited significantly higher photostability than Nile Red. The higher stability of TFMAQ derivatives is attributed to their non-planar (twisted) structure compared with the planar Nile Red structure. Furthermore, the cytotoxicity of compounds **3**–**5** was evaluated using HeLa cells (Fig. S9). At higher concentration (100 μM) of compounds **3**–**5**, MTS assay showed cell viability of over 80%, which demonstrates that the compounds have low cell cytotoxicity. These evaluation results prove that compounds **3** and **5** are valuable fluorescence probes for the live imaging of LDs in cultured cells owing to their high photostability and low cytotoxicity.

## Conclusion

New substituted TFMAQ-8Ar (**3**−**5**) derivatives were synthesized selectively with high yield via C-H activation reaction catalysed by Pd(II). We also achieved selective synthesis of the corresponding TFMAQ-7*N*-arylation product by the appropriate combination of phosphine ligand and palladium source. The TFMAQ-8Ar compounds exhibited fluorescence emissions not only in solution but also in the solid state. In the solutions, compounds **3**−**5** showed fluorescence solvatochromism with high quantum yield in non-polar solvents and considerably lower quantum yield in polar solvents. By this lipophilic-sensitive property, compounds **3** and **5** displayed specific fluorescence imaging of LDs in live cells. In recent years, lipid-related research has attracted increased attention and is actively conducted. The TFMAQ-8Ar derivatives developed in this study are expected to be a versatile fluorescence probe for LDs because of their short synthesis process as well as their high yields and high sensitivity in non-polar environments.

## Methods

### General synthesis of TFMAQ-8Ar compounds

1,1′-Bis(diphenylphosphino)ferrocene palladium(II) dichloride (18 mol%) and potassium *tert*-butoxide (35 mg, 0.28 mmol) were added to a solution of TFMAQ-Ph (**1**, 0.07 mmol) in dry toluene, and this solution was degassed under nitrogen atmosphere. The reaction mixture was heated and stirred at 110 °C for 3 h after the corresponding aryl bromide was added. The reaction mixture was cooled to room temperature, filtered through celite, and extracted with diethylether. The organic layer was dried over Mg_2_SO_4_, filtered, and evaporated in vacuo. The resulting residue was purified by silica-gel column chromatography (*n*-hexane/ethyl acetate) to obtain each TFMAQ-8Ar compound.

### Single crystal X-ray diffraction

The X-ray single crystal structural analyses were performed by same method as our literature^40^. Briefly, all the X-ray reflection data of crystals were collected on a Rigaku R-AXIS RAPID II diffractometer with graphite monochromated Cu Kα radiation (λ = 1.54187 Å) at 93 ± 1 K. The molecular structures were solved using direct methods (SHELXL 97)^41^. The refinements were performed using the full-matrix least squares method from the Crystal Structure software package^42^ to give *P*2_1_/*c* (no. 14), *P*-1 (no. 2), and *C*2/*c* (no. 15) for TFMAQ-8Ph (**3**), TFMAQ-8-t-BuPh (**4**), and TFMAQ-8-OMePh (**5**), respectively. The crystallographic data f in this paper have been deposited with the Cambridge Crystallographic Data Center (CCDC) as nos. CCDC 1936755, 1936762, and 1936763 for compounds **3**, **4**, and **5**, respectively. The structural analyses shown in Table 2 were performed by Mercury software from CCDC.

### Absorption and emission spectra and fluorescent quantum yield measurements

The solution sample (50 μM) for UV-Vis and fluorescence spectra were prepared in each solvent, and nitrogen gas was bubbled before the measurements. The fluorescence quantum yields were calculated from fluorescence spectra measured in a calibrated integrating sphere system as absolute quantum yields.

### Cell culture and fluorescence imaging

3T3-L1 cells were purchased from JCRB (Japanese Collection of Research Bioresources) Cell Bank. 3T3-L1 cells were grown in Dulbecco’s Modified Eagle Medium (DMEM) with 10% Fetal Bovine Serum, 100 units/ml penicillin, and 100 μg/ml streptomycin in a humidified atmosphere of 5% CO_2_ at 37 °C. The cells were grown in 35 mm glass-based dishes, and differentiated by adding 3-isobutyl-1-methylxanthine (0.5 mM), insulin (1.7 μM), and dexamethasone (0.25 μM) to culture media incubated in a CO_2_ incubator for six days. The compounds (**3**–**5**) in DMSO solutions were diluted in culture media (1 μM) and incubated for 30 min before fluorescence imaging. The bright field and fluorescence images were captured by confocal laser microscopy (Nikon, A1R). Excitation laser wavelength at 403 nm and emission wavelength at 525 nm were used for fluorescence imaging of each compound.

## Supplementary Information

Supplementary information
