# Supplementary material for: Selective synthesis of substituted amino-quinoline derivatives by C-H activation and fluorescence evaluation of their lipophilicity-responsive properties
Source: Sci Rep. 2019 Nov 27;9:17723. doi: 10.1038/s41598-019-53882-z (PMC6881333; doi:10.1038/s41598-019-53882-z)

Supporting Information

Selective synthesis of substituted amino-quinoline derivatives by C-H activation and fluorescence evaluation of their lipophilicity-responsive properties

Yasufumi Fuchi,*[a] Masaomi Sakuma,[a] Kohei Ohyama,[a] Ryusuke Hagihara,[b] Minaki Kohno,[a] Koichi Hamada,[a] Akihiro Mizutani,[a] and Satoru Karasawa *[a, c]

[a]*Faculty of Pharmaceutical Sciences, Showa Pharmaceutical University, 3-3165 Higashi-tamagawagakuen, Machida 194-8543, Japan.*

E-mail: fuchi@ac.shoyaku.ac.jp, karasawa@ac.shoyaku.ac.jp, Tel.: +81-42-721-1553

[b]*Graduate School of Pharmaceutical Sciences, Kyushu University, 3-1-1 Maidashi, Higashi-Ku, Fukuoka 812-8582, Japan.*

[c]*PRESTO, Japan Science and Technology Agency, Kawaguchi 332-0012, Japan.*

List of contents

Experimental Procedures p. 2-4
Reaction screening for TFMAQ-Ar derivatives (Table S1) p. 5
Crystallographic and Refinement Parameters of compound **3-5** (Table S2) p. 6
ORTEP drawings of compounds **3-5** (Figure S1) p. 7
UV-Vis and fluorescence spectra of compound **4** and **5** (Figure S2 and S3) p. 8
Fluorescence lifetime and calculated k_{nr} values (Table S3) p. 9
Solid state emission spectra of compounds **3-5** (Figure S4, Table S4) p. 10
Molecular orbitals of compound **3-5** calculated by TD-DFT (Figure S5) p. 11
Cartesian coordinates of compound **3-5** (Table S5) p. 12-14
Co-staining of compound **3** with organellar fluorescence probes (Figure S6) p. 15
3T3-L1 cell imaging after differentiation induction (Figure S7) p. 16
Evaluation of photostability (Figure S8) p. 17
Cell viability assay (Figure S9) p. 18
^{1}H, ^{13}C-NMR spectra of compounds p. 19-21

Experimental procedures

General Information

The ^{1}H and ^{13}C NMR spectra were measured by a Bruker Biospin AVANCE III 300 spectrometer using $CDCl_3$ as the solvent. Infrared spectra for tableting samples with KBr were recorded using a JASCO FT-IR spectrometer. High-resolution electrospray ionization mass spectra were recorded using a JEOL JMS-T 100LP spectrometer. UV−Vis spectra were recorded using JASCO V570 and V760 spectrometers. The fluorescence spectra and quantum yields were recorded on a JASCO FP-8500 spectrofluorimeter equipped with a calibrated integrating sphere system. The fluorescence decay plots were recorded by HORIBA FluoroCube.

Compounds

TFMAQ-8Ph (**3**) The compound was obtained as yellow solid (88%) and recrystallized by *n*-hexane/ethyl acetate for X-ray crystal structure analysis. ^{1}H-NMR ($CDCl_3$, 600 MHz): δ 8.06 (dd, J= 1.8, 9.4 Hz, 1H), 7.83 (d, J= 9.5 Hz, 1H), 7.74 (s, 1H), 7.55 (t, J= 7.5 Hz, 2H), 7.49 - 7.44 (m, 3H), 7.35 (t, J= 7.9 Hz, 2H), 7.15 (d, J= 7.6 Hz, 2H), 7.12 (t, J= 7.4 Hz, 1H), 6.13 (s, 1H). ^{13}C-NMR ($CDCl_3$, 150 MHz): δ 148.19, 147.31 (q, *J*= 35.9 Hz), 146.17, 144.24, 140.95, 136.07 (q, *J*= 31.9 Hz), 134.29, 131.26, 129.79, 129.46, 129.05, 128.18, 124.74, 124.19, 121.72, 121.06, 118.46, 117.20, 110.65. IR (cm^{-1}): 3380, 1598, 1498. ESI-HRMS: Calcd. for [$C_{23}H_{14}F_6N_2$] 433.1139 [M+H]$^+$, Found 433.1111.

TFMAQ-8-t-BuPh (**4**) The compound was obtained as yellow solid (77%) and recrystallized by lyophilisation with DMSO for X-ray crystal structure analysis. ^{1}H-NMR ($CDCl_3$, 600 MHz): δ 8.04 (dd, *J*= 1.6, 9.4 Hz, 1H), 7.81 (d, *J*= 9.4 Hz, 1H), 7.73 (s, 1H), 7.55 (dd, *J*= 1.7, 6.7 Hz, 2H), 7.39 (d, *J*= 8.3 Hz, 2H), 7.35 (t, *J*= 7.9 Hz, 2H), 7.17 (d, *J*= 7.7 Hz, 2H), 7.12 (t, *J*= 7.4 Hz, 1H), 6.22 (s, 1H), 1.41 (s, 9H). ^{13}C-NMR ($CDCl_3$, 150 MHz): δ 150.92, 148.23, 147.19 (q, *J*= 34.5 Hz), 144.36, 141.10, 136.04 (q, *J*= 31.5 Hz), 131.01, 130.86, 129.76, 125.92, 124.71, 124.14, 123.69, 121.84, 121.06, 120.28, 118.46, 110.60, 34.86, 31.51. IR (cm^{-1}): 3388, 2964, 1592, 1503. ESI-HRMS: Calcd. for [$C_{27}H_{22}F_6N_2$] 489.1765 [M+H]$^+$, Found 489.1801.

TFMAQ-8-OMePh (**5**) The compound was obtained as yellow solid (80%) and recrystallized by *n*-hexane/methanol for X-ray crystal structure analysis. ^{1}H-NMR ($CDCl_3$, 600 MHz): δ8.03 (dd, *J*= 1.9, 9.5 Hz, 1H), 7.81 (d, *J*= 9.4 Hz, 1H), 7.72 (s, 1H), 7.37 (t, *J*= 8.8 Hz, 2H), 7.34 (t, *J*= 8.0 Hz, 2H), 7.15 (d, *J*= 8.6 Hz, 2H), 7.11 (t, *J*= 8.4 Hz, 1H), 7.08 (d, *J*= 8.7 Hz, 2H), 6.17 (s, 1H), 3.90 (s, 3H). ^{13}C-NMR ($CDCl_3$, 150 MHz): δ 159.42, 148.34, 147.21 (q, *J*= 35.6 Hz), 144.35, 141.08, 136.06 (q, *J*= 32.1 Hz), 132.47, 129.78, 127.87, 126.13, 124.56, 124.03, 123.66, 122.42,

122.09, 121.49, 121.06, 120.27, 118.53, 114.51, 110.62, 55.44. IR (cm^{-1}): 3397, 2922, 1592, 1504. ESI-HRMS: Calcd. for [$C_{24}H_{16}F_6N_2O$] 485.1065 $[M+Na]^+$, Found 485.1096.

*Screening of arylation reaction for TFMAQ-Ph **1***

Palladium(II) catalyst (18 mol%), phosphine ligand (30 mol%) and base (0.21 mmol) were added to a solution of TFMAQ-Ph (**1**, 0.07 mmol) in solvent, and this solution was degassed under nitrogen atmosphere. The reaction mixture was heated and stirred at 110 °C for 3 h after the corresponding aryl bromide was added. The reaction mixture was cooled to room temperature, filtered through celite and extracted with diethylether. The organic layer was dried over $MgSO_4$, filtered and evaporated in vacuo. The resulting residue was purified by preparative TLC (*n*-hexane/ethyl acetate) to obtain TFMAQ-7,7-diPh or TFMAQ-7,8-diPh compounds.

Fluorescence lifetime measurements

The solution sample (50 μM) were prepared in each solvents and nitrogen gas was bubbled before the measurements. The fluorescence decay plots were collected by HORIBA FluoroCube, and analyzed by Das Analysis (HORIBA) to calculate the τ values by linear fitting with first-order exponential decay under 1.2 of chi-square.

DFT Calculation

Geometries of the studied molecules were optimized at the B3LYP/6-31++G** level of theory using the Gaussian 09 program[s1]. TDDFT calculations of the studied molecules were performed at the same level of theory. All calculations were performed by non-solvation model.

Cell culture and fluorescence imaging of Hela cells

HeLa cells were purchased from JCRB (Japanese Collection of Research Bioresources) Cell Bank. HeLa cells were grown in Dulbecco's Modified Eagle Medium (DMEM) with 10% Fetal Bovine Serum (FBS), 100 units/ml penicillin and 100 μg/ml streptomycin (GIBCO, Invitrogen) in a humidified atmosphere of 5% CO_2 at 37 °C. The cells were grown to confluence in 35 mm glass-based dishes, washed with PBS buffer and treated with 50 μM of compound **3** and 50 nM of MitoRed (DOJINDO Molecular Technologies) or 50 nM of LysoTracker-Deep Red (Thermo Fisher Scientific) in DMEM. After incubation for 30 min in a CO_2 incubator, the dish was washed with medium two times. The fluorescence signals from the live cells were captured by confocal laser microscopy (Nikon A1R). Excitation laser wavelength: 403 nm for sensor **3**, 590 nm for MitoRed and 640 nm for LysoTracker-Deep Red.

MTS assay

HeLa cells were dispensed into 96-well plate by 10,000 cells/well and incubated at 37°C overnight. The compounds **3-5** in DMSO were diluted with culture medium (100 μL/well) at several concentrations (n= 3) in above-mentioned wells. The prepared plates were incubated at 37 °C for 2h, washed with DMEM twice and replaced to fresh DMEM containing FBS. This plates were incubated with CellTiter 96 (Promega, 20 μL/well) for 4 h at 37 °C before absorbance at 490 nm in each wells were measured by Varioskan Flash (Thermo Scientific). The ratio of absorbance with non-treated wells were plotted as cell viability rates.

Evaluation of photostability

The compounds in methanol (20 μM, 2 mL) solution were placed into the quartz cell for a fluorescence spectrometer with stirring bar. They were magnetically stirred on the stirrer and irradiated by a high pressure Hg lamp (1000w, Ushio) through a collecting lens. The reactions were followed by absorption spectrometry. The absorbance at 430 nm (for TFMAQ-8Ar compounds) and 580 nm (for Nile Red) were plotted as function of irradiation time (1, 3, 5, 10, 20, 30, 40, 50 and 60 min).

Reference)

S1) M. J. Frisch, G. W. Trucks, H. B. Schlegel, G. E. Scuseria, M. A. Robb, J. R. Cheeseman, G. Scalmani, V. Barone, B. Mennucci, G. A. Petersson, H. Nakatsuji, M. Caricato, X. Li, H. P. Hratchian, A. F. Izmaylov, J. Bloino, G. Zheng, J. L. Sonnenberg, M. Hada, M. Ehara, K. Toyota, R. Fukuda, J. Hasegawa, M. Ishida, T. Nakajima, Y. Honda, O. Kitao, H. Nakai, T. Vreven, J. A. Montgomery, Jr., J. E. Peralta, F. Ogliaro, M. Bearpark, J. J. Heyd, E. Brothers, K. N. Kudin, V. N. Staroverov, T. Keith, R. Kobayashi, J. Normand, K. Raghavachari, A. Rendell, J. C. Burant, S. S. Iyengar, J. Tomasi, M. Cossi, N. Rega, J. M. Millam, M. Klene, J. E. Knox, J. B. Cross, V. Bakken, C. Adamo, J. Jaramillo, R. Gomperts, R. E. Stratmann, O. Yazyev, A. J. Austin, R. Cammi, C. Pomelli, J. W. Ochterski, R. L. Martin, K. Morokuma, V. G. Zakrzewski, G. A. Voth, P. Salvador, J. J. Dannenberg, S. Dapprich, A. D. Daniels, O. Farkas, J. B. Foresman, J. V. Ortiz, J. Cioslowski, and D. J. Fox, *Gaussian 09, Revision D.01*, Gaussian, Inc., Wallingford CT, 2013.

Table S1 Reaction screening for TFMAQ-8Ar derivatives.

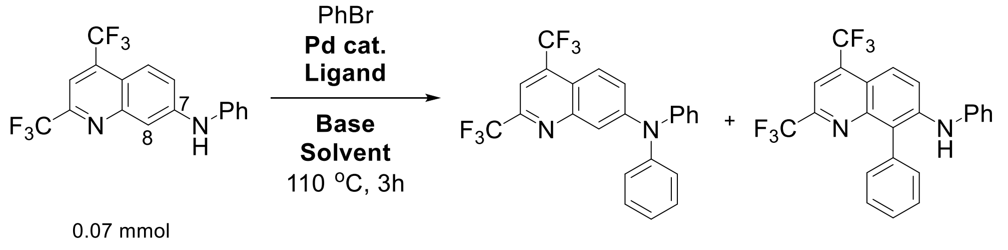

Entry	[Pd]	Ligand	Base	Solvent	2 yield (%)	3 yield* (%)
1	$Pd(OAc)_2$	DPPF	K_2CO_3	DMF	n.d.	n.d.
2	$Pd(OAc)_2$	DPPF	*t*-BuOK	DMF	n.d.	n.d.
3	$Pd(OAc)_2$	DPPF	K_2CO_3	toluene	n.d.	n.d.
4	$Pd(OAc)_2$	DPPF	Ag_2CO_3	toluene	Trace	Trace
5	$Pd(OAc)_2$	DPPF	Cs_2CO_3	toluene	Trace	Trace
6	$Pd(OAc)_2$	DPPF	*t*-BuOK	toluene	Trace	40

*The yield was determined by ^{1}H-NMR.

Table S2 Crystallographic and Refinement Parameters of Single-Crystal Analyses of compound **3-5**

	**3**	**4**	**5**
**Empirical formula**	$C_{23}H_{14}F_6N_2$	$C_{27}H_{22}F_6N_2$	$C_{24}H_{16}F_6N_2O$
**Formula weight**	432.37	488.47	462.39
**Crystal dimensions**	0.5 x 0.2 x 0.1 mm	0.3 x 0.3 x 0.2 mm	0.4 x 0.3 x 0.2 mm
**Crystal system**	monoclinic	monoclinic	triclinic
**Space group**	$P2_1/c$ (#14)	$C2/c$ (#15)	P-1 (#2)
***a* / Å**	23.0031(7)	37.0177(9)	7.0649(2)
***b* / Å**	9.3968(3)	13.9753(4)	12.7619(3)
***c* / Å**	8.5079(2)	19.0543(5)	13.0219(3)
**α / deg**	90	90	111.958(8)
**β / deg**	94.418(6)	106.315(8)	99.050(7)
**γ / deg**	90	90	103.997(8)
**Volume / Å^3**	1833.6(1)	9460.5(6)	1015.7(1)
Z	4	16	2
**Density (calc.) / g/cm^3**	1.566	1.372	1.512
F_{000}	880.00	4032.00	472.00
**$2\theta_{max}$ cutoff / deg**	136.4	136.4	136.4
**Reflections collected**	19711	50355	11583
**Unique reflections**	3313	8513	3646
R_{int}	0.1233	0.0905	0.0774
**Absorption coefficient / cm^{-1}**	11.812	9.774	11.442
**R_1 [I>2.00σ(I)]**	0.0563	0.0645	0.0548
**wR_2 (All reflections)**	0.1325	0.1614	0.1471
**Goodness-of-fit on F^2**	0.991	1.051	1.048
**Largest diff. peak/hole / e Å^{-3}**	0.36/-0.23	0.48/-0.36	0.67/-0.66
**CCDC No.**	**1936755**	**1936762**	**1936763**

Figure S1 ORTEP drawings of compounds **3–5** with 50% ellipsoid probability

TFMAQ-8Ph (**3**)

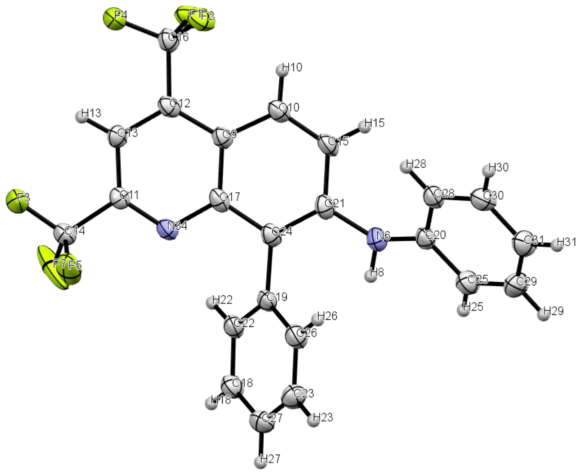

TFMAQ-8tBuPh (**4**)

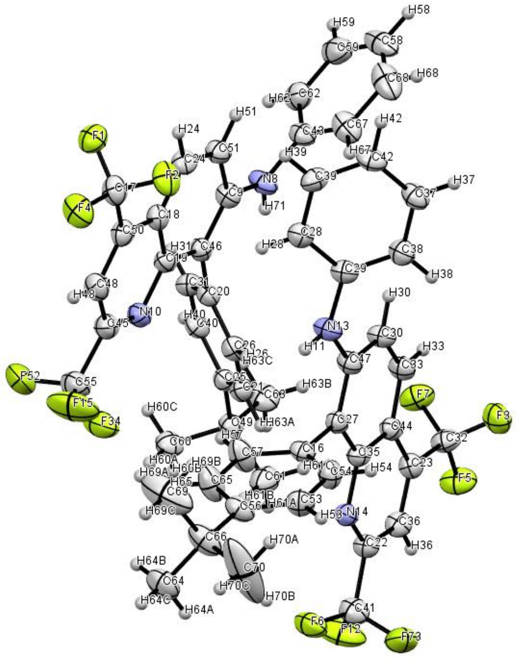

TFMAQ-8MeOPh (**5**)

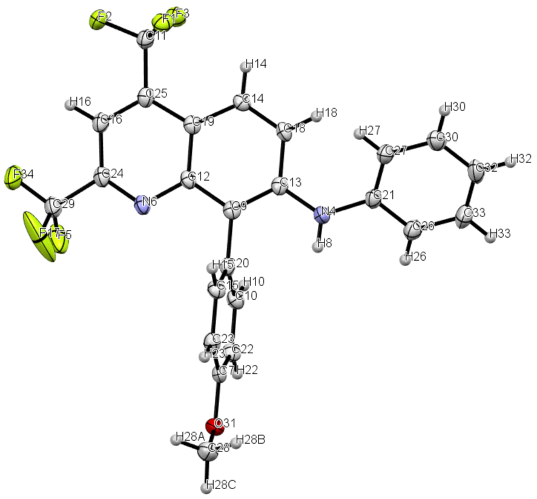

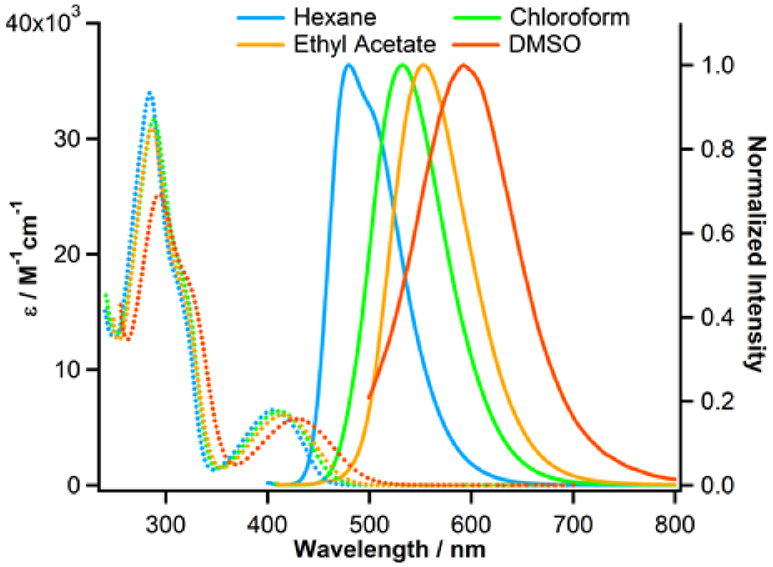

Figure S2 UV-Vis absorption (dotted line) and fluorescence emission (solid line) spectra of compound **4** in various solution.

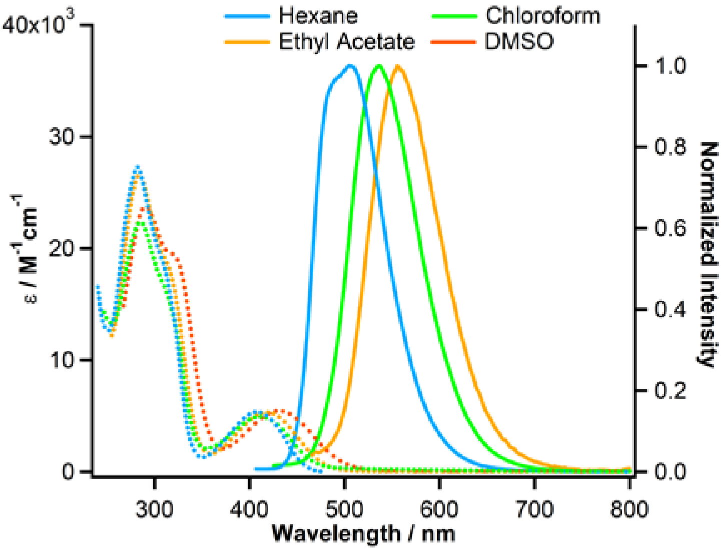

Figure S3 UV-Vis absorption (dotted line) and fluorescence emission (solid line) spectra of compound **5** in various solution. The emission spectrum in DMSO was not detected.

Table S3 Fluorescence lifetime and calculated k_{nr} values of TFMAQ-8Ar compounds in each solvent.

	solvent	Φ_{fl}	τ / ns	k_r / ns^{-1}	k_{nr} / ns^{-1}
**1**[a]	Hex	0.48	8.92	0.054	0.058
	$CHCl_3$	0.32	9.06	0.035	0.089
	AcOEt	0.01	0.62	0.016	1.6
	DMSO	<0.01	-	-	-
**3**	Hex	0.51	14.8	0.034	0.033
	$CHCl_3$	0.54	8.66	0.062	0.053
	AcOEt	0.03	1.27	0.024	0.764
	DMSO	n.d.	-	-	-
**4**	Hex	0.38	15.6	0.024	0.040
	$CHCl_3$	0.33	11.3	0.029	0.059
	AcOEt	0.06	2.71	0.022	0.347
	DMSO	0.01	-	-	-
**5**	Hex	0.57	16.7	0.034	0.026
	$CHCl_3$	0.42	13.0	0.032	0.045
	AcOEt	0.12	5.13	0.023	0.172
	DMSO	n.d.	-	-	-

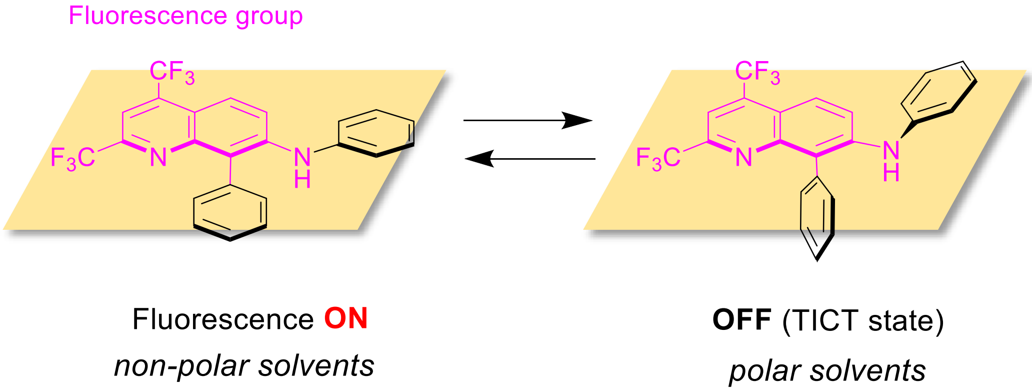

Scheme S1 Proposed structural mechanism of fluorescence quenching by TICT.

A)

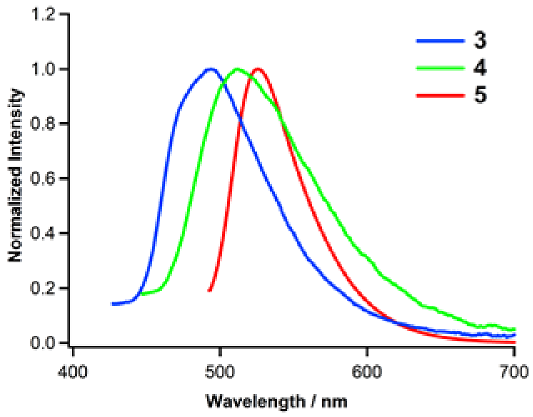

B)

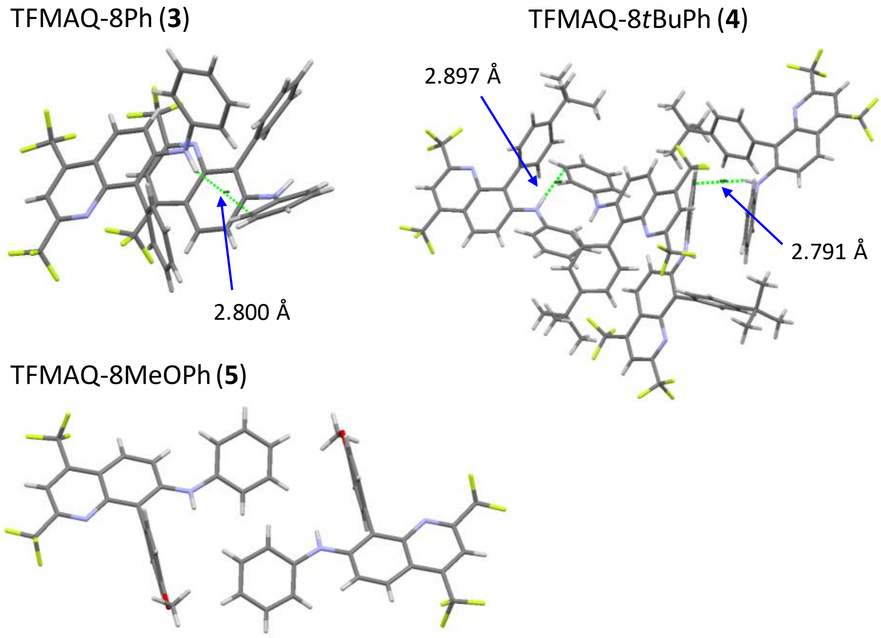

Figure S4 A) Normalized emission spectra of compound **3**, **4** and **5** in the solid state (Excitation wavelength: 400 nm). **B)** Intermolecular NH-π interaction within the crystal packing.

Table S4 Photophysical data of compound **1**, **3**, **4** and **5** in the solid state.

	λ_{abs} (nm)	λ_{FL} (nm)	Φ_{fl}
**1**	425	464	0.28
**3**	404	493	0.11
**4**	419	512	0.19
**5**	426	526	0.38

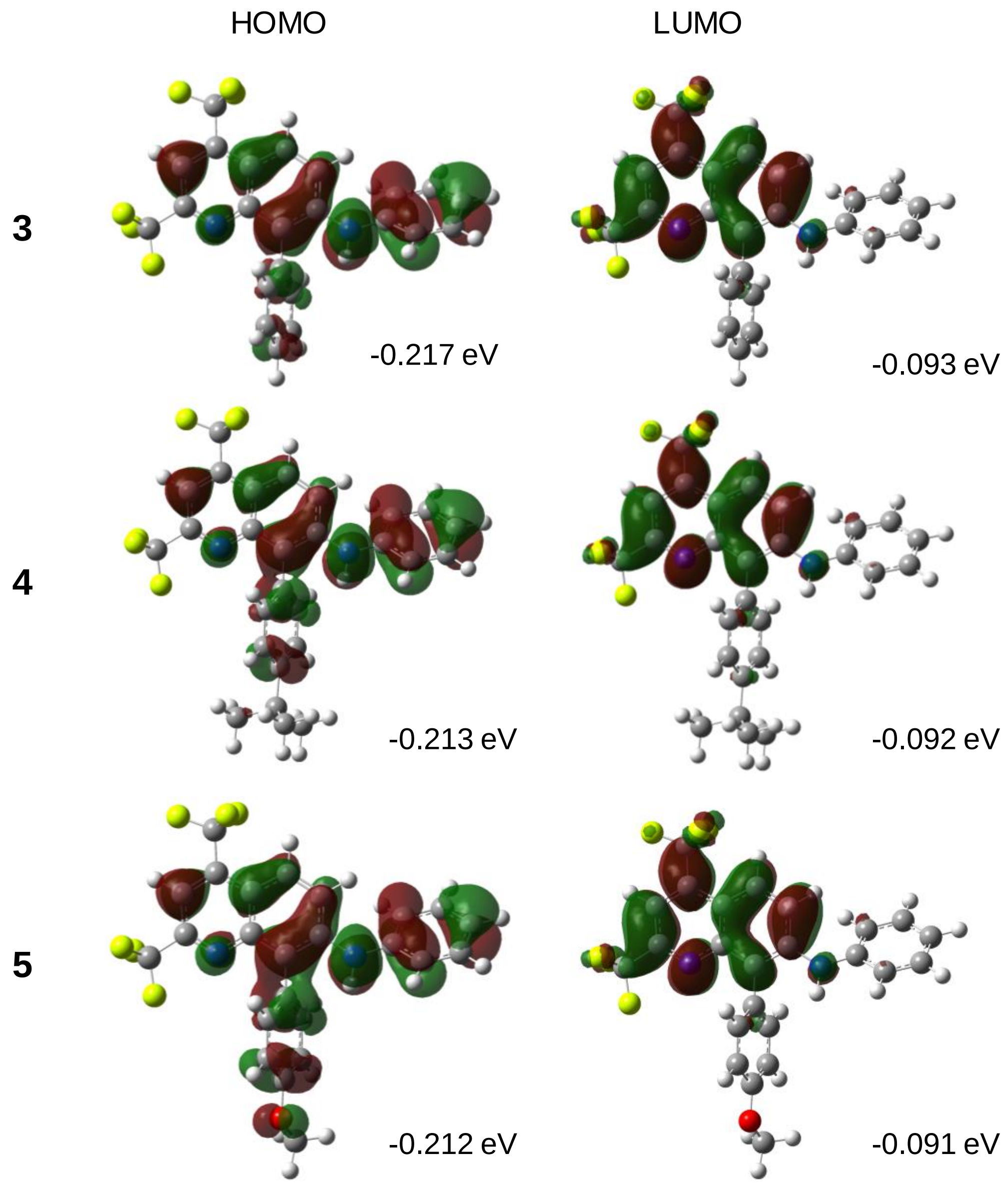

Figure S5 Molecular orbitals of compound **3-5** at excited state calculated by TD-DFT method at the basis level of B3LYP/6-31G++ (d, p) using Gaussian 09 program.

Table S5 Cartesian Coordinates

The cartesian coordinates (in Å) of compound **3-5** optimized at the B3LYP/6-31++G** level.

Compound **3**

C	-2.99819400	0.53148100	0.08106100
C	-0.74575700	0.07945900	-0.07800800
C	-0.99141700	-1.33732100	-0.12769800
C	-2.34836700	-1.76331500	-0.06627300
C	-3.36120700	-0.83520800	0.03673100
C	0.58300800	0.59315300	-0.11968200
C	0.12781900	-2.20473700	-0.24268800
H	-4.40011400	-1.13475000	0.08116000
C	1.40326900	-1.70332700	-0.29119400
C	1.65958900	-0.30132000	-0.21785500
H	-0.01731900	-3.27569200	-0.31217900
H	2.23611900	-2.38444000	-0.41842000
C	0.82414800	2.06735900	-0.07420500
C	1.38579500	2.66274900	1.06755600
C	0.51039500	2.88258300	-1.17332700
C	1.62237300	4.03888100	1.11106400
H	1.62539600	2.04444700	1.92835800
C	0.74778600	4.25796400	-1.13079800
H	0.06714400	2.43580900	-2.05820600
C	1.30448100	4.84016400	0.01121100
H	2.04873600	4.48376600	2.00559200
H	0.49179200	4.87464000	-1.98750800
H	1.48528700	5.91053500	0.04542600
N	-1.76815400	0.97872700	0.02991400
N	2.95143500	0.18697600	-0.32372200
H	3.02125100	1.17811000	-0.51248300
C	4.17225300	-0.47956900	-0.09275700
C	5.29500900	-0.09658500	-0.84603300
C	4.32012100	-1.46243500	0.90032500
C	6.53652400	-0.68748400	-0.61368800
H	5.18435600	0.65982500	-1.61840700
C	5.56241000	-2.06373100	1.11001900
H	3.47471200	-1.73779900	1.52166700
C	6.67699800	-1.68247100	0.35831100
H	7.39334900	-0.37709800	-1.20459500
H	5.66003800	-2.82208500	1.88157100
H	7.64160700	-2.14913400	0.53129200
C	-2.70059900	-3.23376600	-0.11650900
C	-4.10473400	1.56578600	0.22606400
F	-2.27825900	-3.81475300	-1.26971500
F	-2.12847500	-3.92645600	0.90243100
F	-4.03230100	-3.44688400	-0.03558100
F	-5.10295600	1.32986300	-0.66911200
F	-3.68378900	2.82297400	0.04462000
F	-4.67261300	1.49348600	1.46109300

Compound **4**

F	4.97344600	1.99843600	0.88823800
F	6.05205800	0.35889500	-0.05420300
F	1.55556700	-4.01674600	0.35585800
F	4.99190000	1.80868800	-1.28548900
F	3.41727900	-3.92394700	-0.77819100
F	3.44889700	-3.69907500	1.39400000
N	-1.55757400	2.37093700	-0.36481900
N	1.39964500	-1.38678000	0.04968900
C	-1.24562900	-0.45282500	-0.09434500
C	2.61045600	-1.88419400	0.12247600
C	3.67360200	0.24840600	-0.06705000
C	-0.07960100	0.47688800	-0.13535400
C	-3.18801900	4.09359200	-0.79555500
C	-2.03855200	3.66794000	-0.10663400
C	0.86093800	2.72601800	-0.32226300
C	4.92566900	1.09628200	-0.12869900
C	2.14134700	2.23787100	-0.27060600
C	1.24796400	-0.03909000	-0.08400500
C	3.79783600	-1.11922400	0.06576700
C	-2.02590400	5.78324700	1.08755900
C	-1.47083600	4.52417700	0.85522000
C	-3.74680200	5.34473300	-0.53952700
C	2.73120600	-3.39236700	0.27515300
C	-3.16512700	6.20374000	0.39626200
C	2.38512400	0.84625500	-0.13793600
C	-0.27347300	1.86544700	-0.23751700
C	-3.28352900	-1.26263600	0.97380200
C	-2.18040900	-0.40341300	0.94911800
C	-3.50688100	-2.20790200	-0.03969800
C	-1.45481900	-1.40061900	-1.10713100
C	-4.14004400	-4.58316400	0.43377300
C	-2.55755100	-2.25342200	-1.07770300
C	-4.68453200	-3.19671800	-0.00334000
C	-5.34560100	-3.33559700	-1.39652300
C	-5.78225300	-2.76286500	0.99126800
H	-2.26900300	1.67235300	-0.53520000
H	-3.63211500	3.43955100	-1.54163300
H	0.70839000	3.78856000	-0.46808000
H	2.96936000	2.93170300	-0.34827600
H	4.76963100	-1.59184300	0.12078500
H	-1.57253300	6.43094700	1.83260200
H	-0.61566200	4.19789000	1.43640200
H	-4.63483300	5.65107300	-1.08485200
H	-3.59452900	7.18203100	0.58794700
H	-3.96795400	-1.18285700	1.81061800
H	-2.03789300	0.30122600	1.76444200
H	-0.73863500	-1.48246000	-1.91956600
H	-3.68095500	-4.52889900	1.42629700
H	-3.37998300	-4.94249000	-0.26763000
H	-4.95242200	-5.31839100	0.46783200
H	-2.66153100	-2.97578600	-1.88025400
H	-4.66394800	-3.74567700	-2.14564000
H	-5.70891500	-2.36870700	-1.76161100
H	-6.19974200	-4.01811100	-1.33412800
H	-6.17552000	-1.76975500	0.74641700
H	-5.42238500	-2.74434700	2.02428800
H	-6.61311700	-3.47471300	0.95414800

Compound **5**

C	-2.99643300	0.86941900	0.07649200
C	-0.93666500	-0.14437100	-0.11064500
C	-1.53631900	-1.45237400	-0.11554600
C	-2.95552400	-1.51747900	-0.01948600
C	-3.69656100	-0.36023500	0.07563900
C	0.47895100	0.01379500	-0.18711700
C	-0.67897100	-2.57973300	-0.22225800
H	-4.77619400	-0.38348800	0.14784800
C	0.68092000	-2.42173900	-0.30197400
C	1.28741000	-1.13082200	-0.27105200
H	-1.09437200	-3.57899100	-0.26106900
H	1.30977400	-3.29566800	-0.42371100
C	1.08763300	1.37687300	-0.19445700
C	0.91315100	2.24732800	-1.28689100
C	1.85603500	1.82874700	0.88580200
C	1.48329800	3.51376300	-1.29714500
H	0.31307600	1.92725100	-2.13307100
C	2.43486200	3.10293700	0.89347100
H	1.99788900	1.18282500	1.74804800
C	2.24805200	3.95182000	-0.20457800
H	1.34263300	4.18550800	-2.13761300
H	3.01341100	3.41618700	1.75429200
N	-1.69507000	0.98729000	-0.01057000
N	2.65952100	-0.99410800	-0.41001300
H	2.97229700	-0.05866800	-0.63460500
C	3.67659800	-1.92847000	-0.13377800
C	4.87237500	-1.84798900	-0.86857900
C	3.56690600	-2.89095800	0.88444400
C	5.93092900	-2.71264900	-0.59356700
H	4.96086700	-1.10863000	-1.66018500
C	4.62494100	-3.76591500	1.13738000
H	2.67031300	-2.93897600	1.49276100
C	5.81162800	-3.68493700	0.40412300
H	6.84711000	-2.63349700	-1.17155400
H	4.52339600	-4.50378900	1.92814000
H	6.63228900	-4.36474500	0.61029500
C	-3.67000200	-2.85098100	-0.02661100
C	-3.80255200	2.15337800	0.20445800
F	-3.45029400	-3.53417100	-1.18033800
F	-3.25625700	-3.65449800	0.98775200
F	-5.00845700	-2.71840600	0.10106700
F	-4.77943700	2.20380600	-0.74287700
F	-3.06207900	3.26105800	0.08526400
F	-4.43515300	2.20518700	1.40905900
O	2.76402900	5.21167600	-0.30744500
C	3.51942700	5.72991900	0.78120900
H	3.81315100	6.73764500	0.48544100
H	2.91702200	5.77986200	1.69667000
H	4.41849400	5.12874200	0.96659200

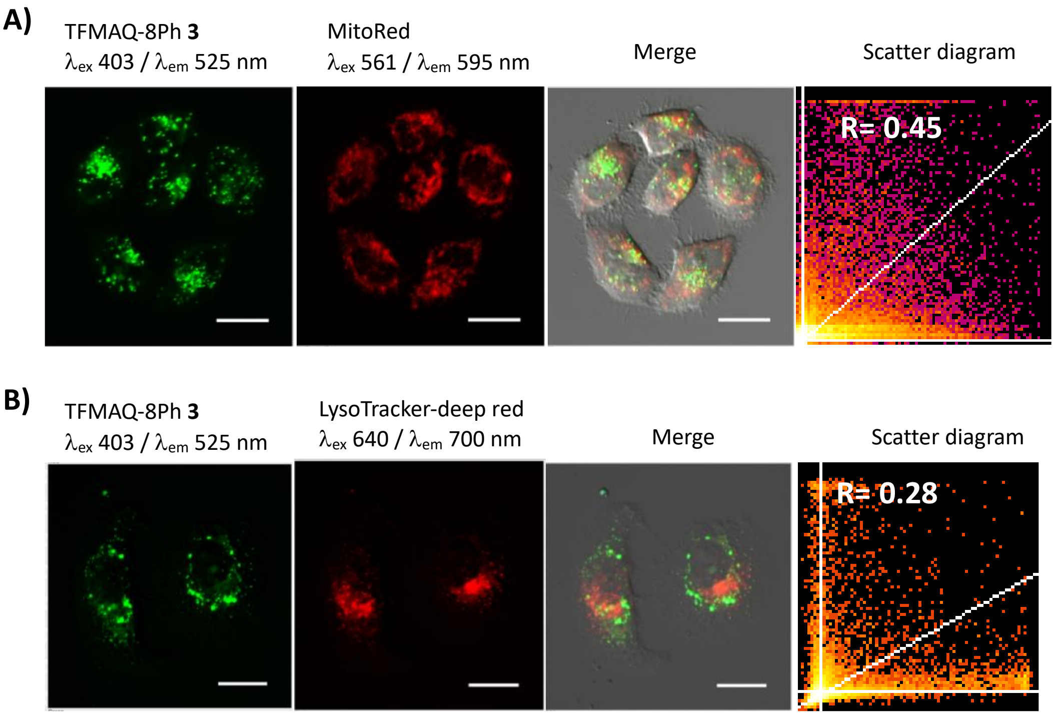

Figure S6 Co-staining of TFMAQ-8-Ph **3** with fluorescence probes for mitochondria or lysosome into Hela cells. **A)** Co-staining with mitochondria probe (Mito Red) **B)** Co-staining with lysosome probe (LysoTracker-deep red). R values mean Pearson's correlation coefficient. Scale bars = 20 μm.

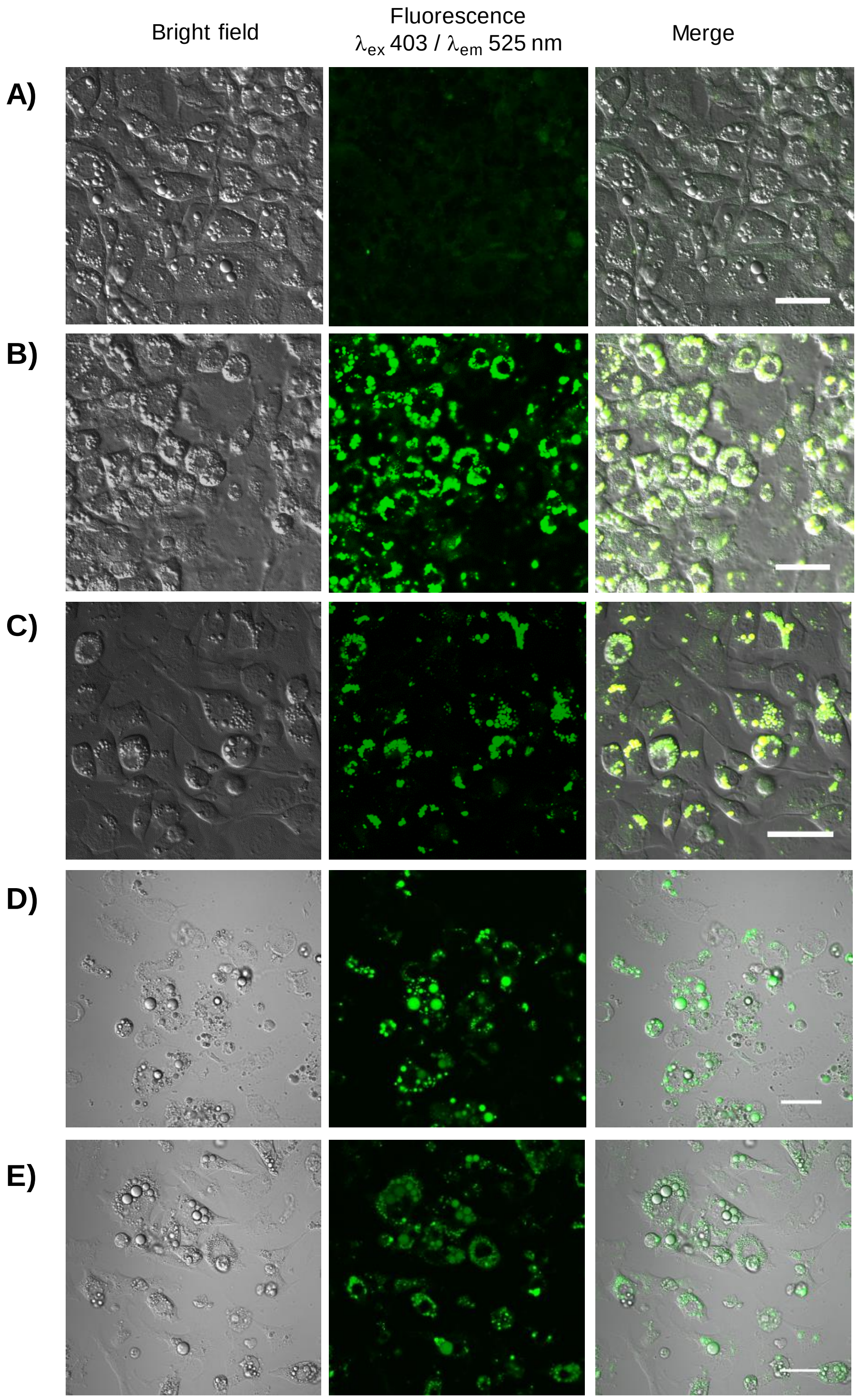

Figure S7 3T3-L1 cell images after differentiation induction by confocal laser microscopy staining with compound **4** or **5** (1 or 10 μM in cultured medium). **A)** Staining with compound **4** (1 μM) **B)** Staining with compound **5** (1 μM) **C)** Staining with compound **4** (10 μM) excited by higher laser intensity. **D)** Staining with compound **3** (0.1 μM) **E)** Staining with compound **5** (0.1 μM). Scale bars = 50 μm.

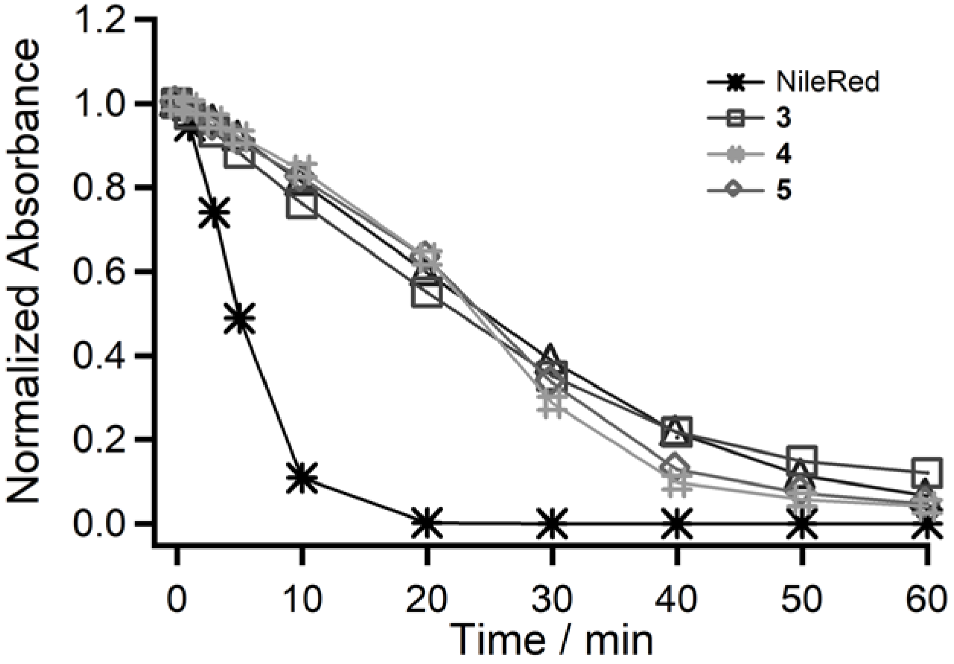

Figure S8 Evaluation of photostability for compound **3-5** and Nile Red (20 μM in MeOH). Normalized absorbance (430 nm for compounds **3-5** and 580 nm for Nile Red) values were plotted at each time after irradiation.

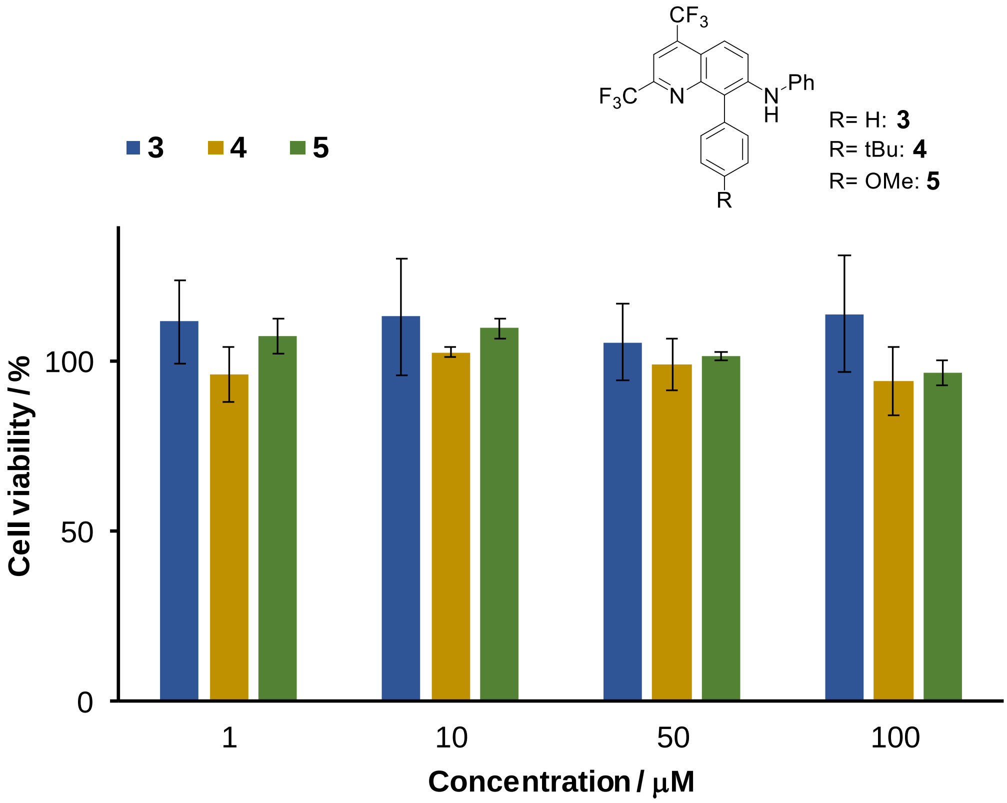

Figure S9 Cell viability assay of compound **3-5** (1-100 µM) for Hela cells using MTS assay method. Cell viabilities (%) were calculated from the absorbance rate at 490 nm for non-treated cells.

^{1}H-NMR spectrum of compound **3**

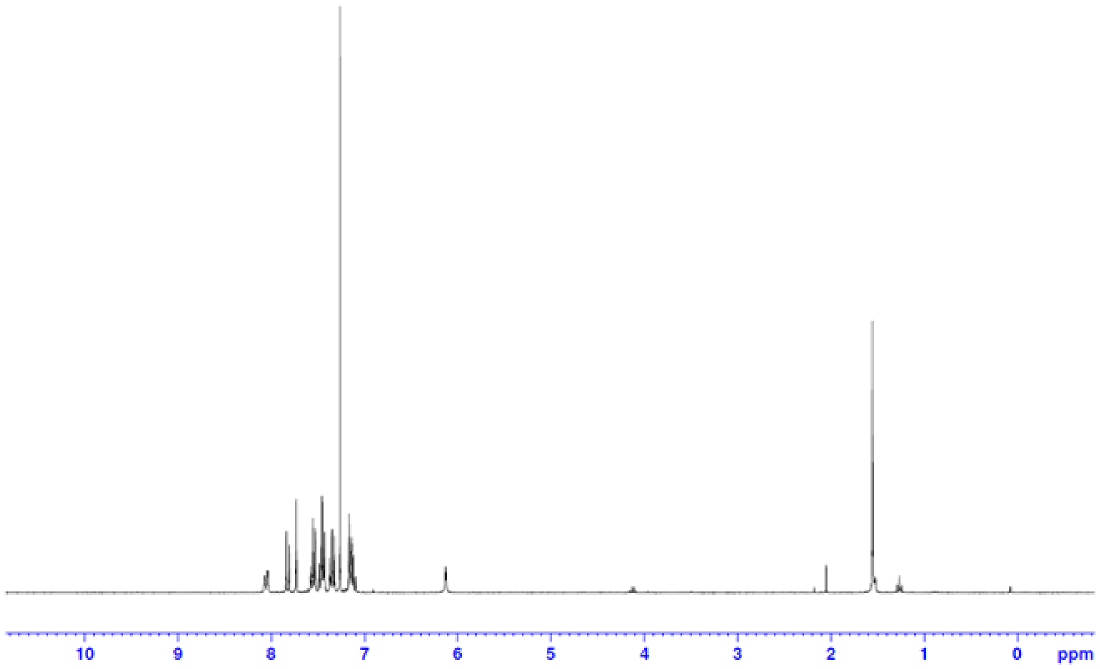

^{13}C-NMR spectrum of compound **3**

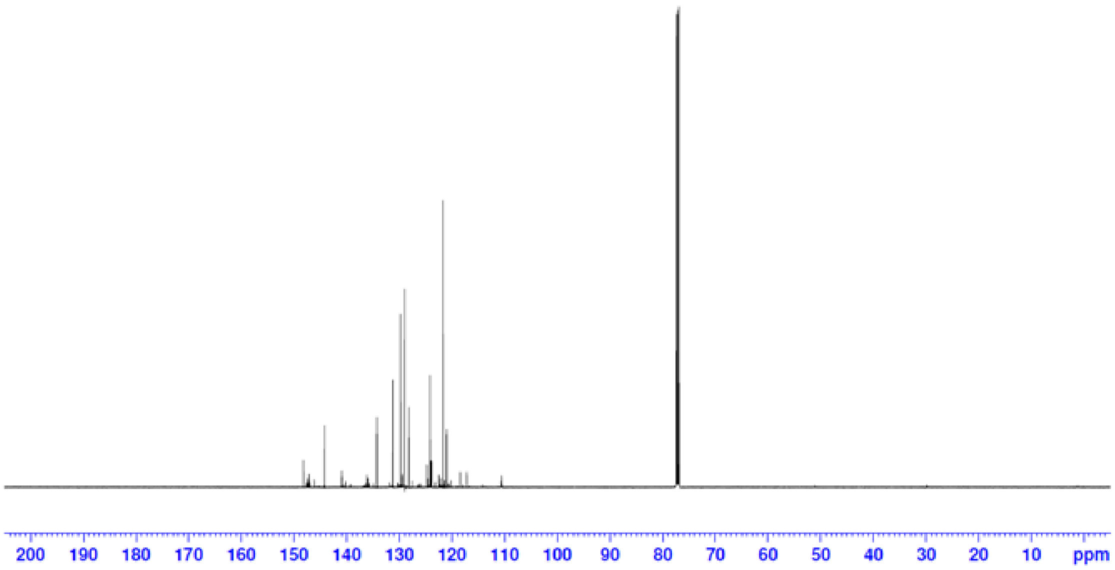

^{1}H-NMR spectrum of compound **4**

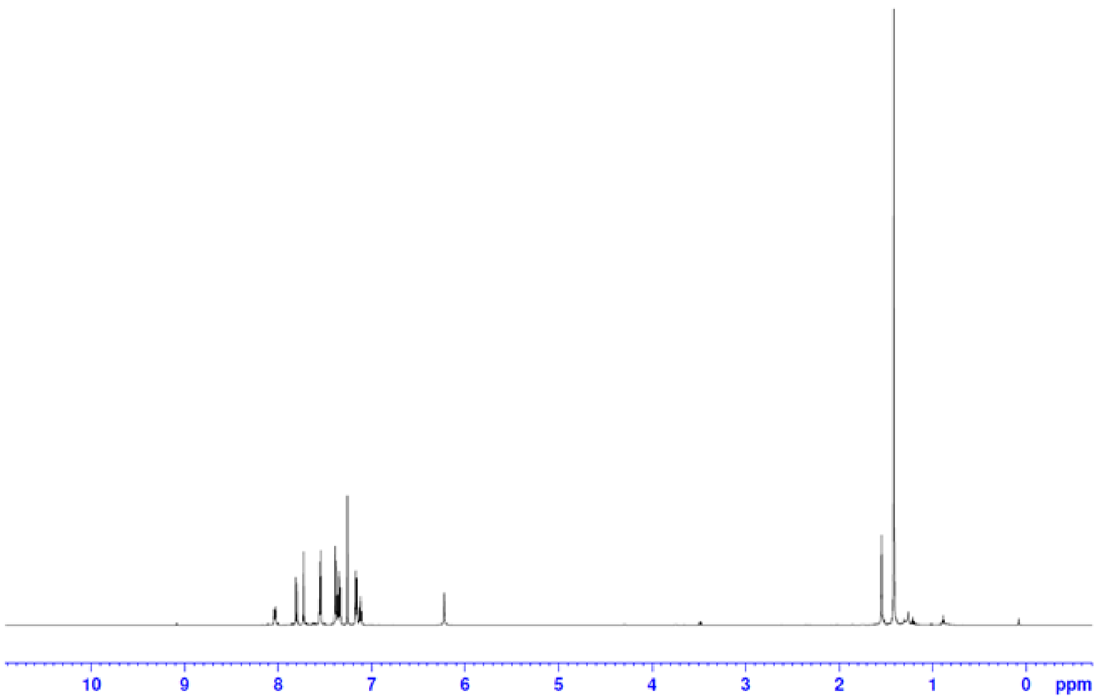

^{13}C-NMR spectrum of compound **4**

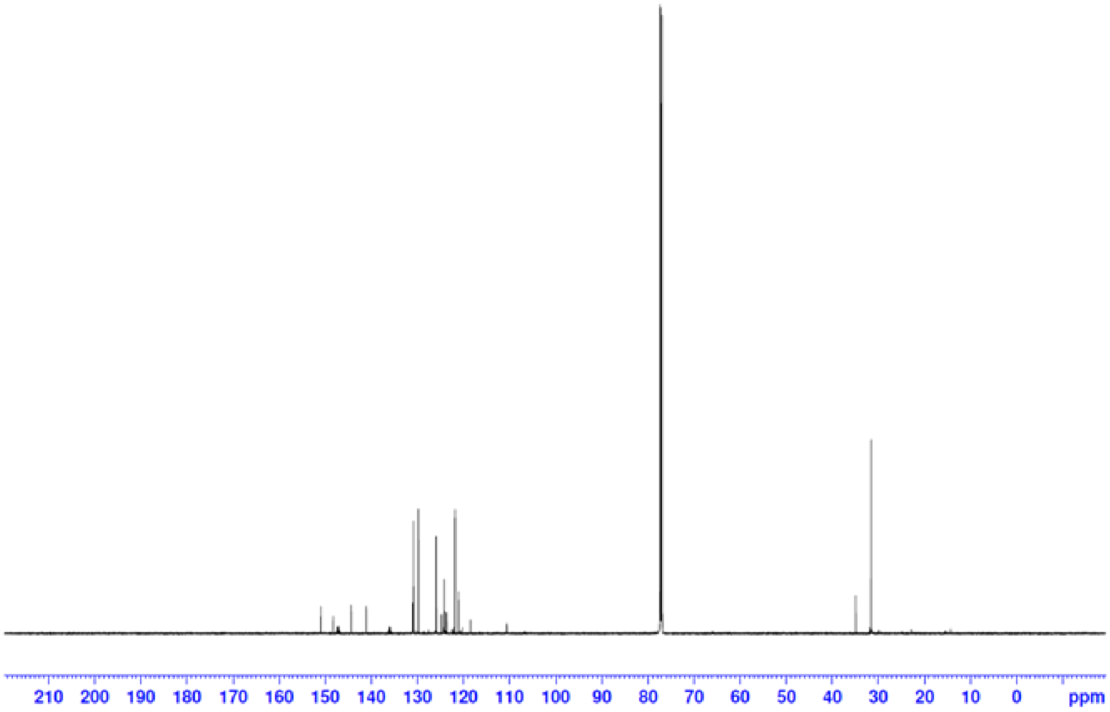

^{1}H-NMR spectrum of compound **5**

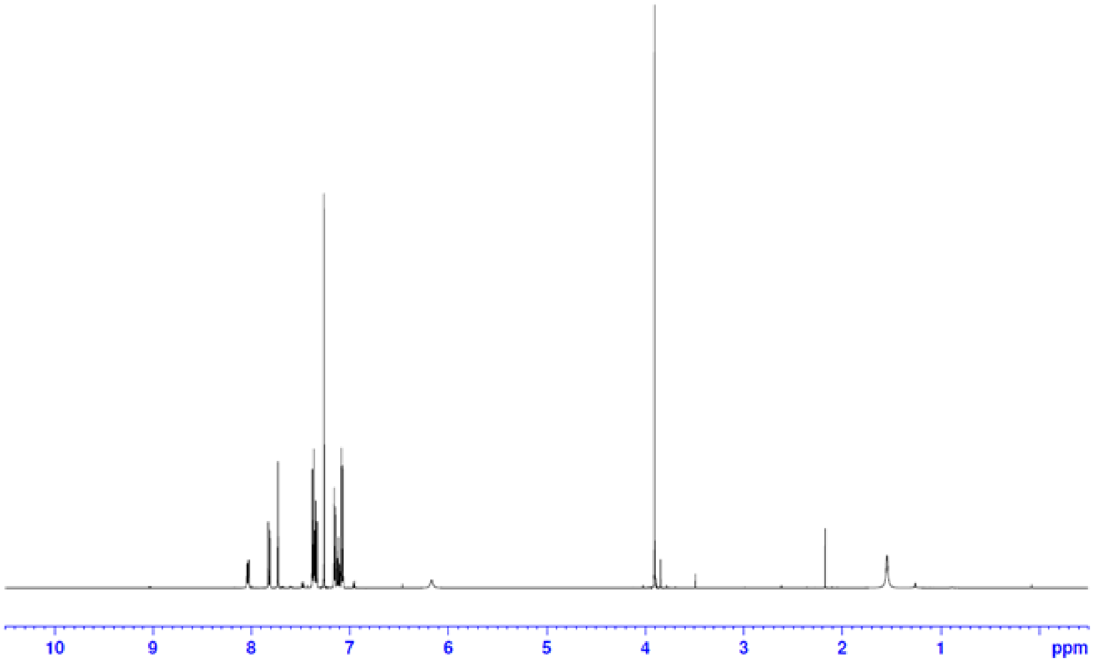

^{13}C-NMR spectrum of compound **5**

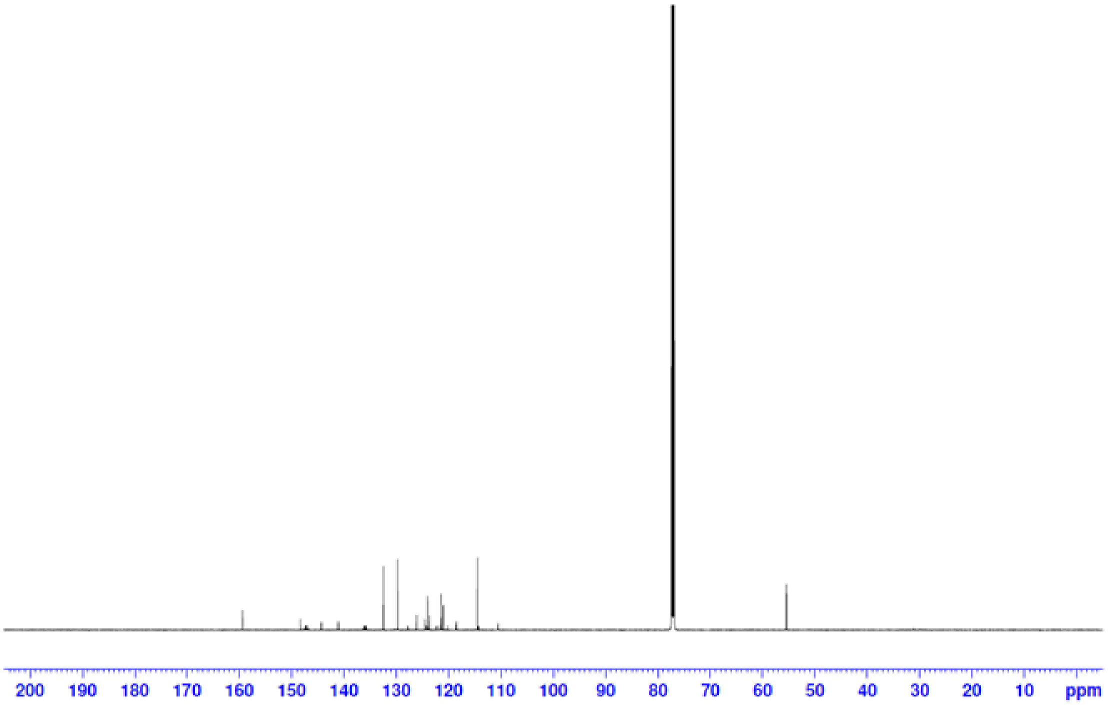

Supplement: Supplementary file 1 — Supplementary information [file 41598_2019_53882_MOESM1_ESM.pdf]
